# Examining the differences between information professional groups in perceiving information ethics: An analytic hierarchy process study

**DOI:** 10.3389/fpsyg.2022.954827

**Published:** 2022-09-27

**Authors:** Hsiu-Ping Yueh, Ching-Yin Huang, Weijane Lin

**Affiliations:** ^1^Department of Psychology, National Taiwan University, Taipei City, Taiwan; ^2^Department of Bio-Industry Communication and Development, National Taiwan University, Taipei City, Taiwan; ^3^Department of Library and Information Science, National Taiwan University, Taipei City, Taiwan

**Keywords:** information ethics, professional ethics, analytical hierarchy process, library information science, information technology - IT

## Abstract

Information and communication technology (ICT) has a great impact on contemporary society and people’s lives. Especially with the pervasive access to rapidly developing technology, the impact of ICT on society and human values, the norms of ICT use, and the ethical issues derived from them are beyond the past ethical framework and deserve more research attention. The purpose of this study was to explore the key factors that influence the decision-making behaviors of information professionals when they are faced with information ethics issues. The study adopted the analytic hierarchical process method to develop the evaluation framework and criteria for information professional ethics and employed the professional fields of library and information science and information technology as examples to compare whether information professionals in different fields make different judgments on the aforementioned decision-making criteria. The results of the study validated the professional information ethics hierarchy and criteria and contributed to the field of information ethics research by providing information on the aspects that need attention in the cultivation of professionals in different fields.

## Introduction

The rapid development of information technology has led to dramatic changes in the economies, politics, and cultures of countries around the world. The Internet now plays a crucial role in a globalized society, but while the Internet provides considerable convenience, it also generates a number of ethical issues. For example, the Internet allows easy access to various materials under copyright protection, which is largely disregarded by many users. Reports on information systems security and control show that this unethical Internet activity has resulted in significant losses of profit for publishing companies (Anthes, 1996). As Mason (1986) stressed, information ethics is critical in the information era and should be emphasized, identified, and classified as a new and specific area of knowledge to be explored and studied (Floridi and Sanders, 2001).

In the general approach to ethical dilemmas, decision makers must choose one of two different ethical values in preference to the other (Maclagan, 2003). Information ethics is concerned with the moral dilemmas and ethical conflicts that arise when human beings interact with information, information and communication technologies (ICTs), and information systems (Carbo, 2008). Critical issues, including privacy, property, accuracy, and accessibility of information, are often encountered by people during their interaction with information (Mason, 1986), and throughout their careers, students who major in information-related fields will face a wide range of ethical dilemmas related to issues such as trust (Kelton et al., 2008) and transparency (Fleischmann and Wallace, 2009). Employees’ lack of information ethics could compromise institutional information security, damage an institution’s reputation, or even cause financial losses. Therefore, for information majors, information ethics is not simply part of their literacy but also directly associated with their profession.

With reference to the importance of information ethics for professional development, the Association for Computing Machinery (ACM, 1992) proposed the ACM Code of Ethics and Professional Conduct, which identifies the personal responsibility and commitment of information professionals. Information ethics also gained attention from the research field of information science education. Chang (2011) utilized Kohlberg’s cognitive moral development model to measure improvements in students’ information ethics values through technology-mediated learning models. The results showed that e-learning materials that involved multiple media representations improved students’ comprehension as well as respect toward the norms, privacy, accessibility, and intellectual property issues.

However, current resources for learning and training of information ethics remain quite limited. According to a local investigation of the undergraduate curriculum by Lin and Chou (2014), only 37% of all the 199 departments or institutes of information science and engineering in Taiwan had courses related to information ethics. For this limited number of courses, their further review of 53 syllabi suggested that most of these courses were taught with didactic approaches in classroom teaching, which failed to convey the dynamic and situated nature of information ethics. Pedagogically, previous studies (Liu and Yang, 2012; Fiesler et al., 2020) supported that the ability to assess ethical dilemmas involved high-level thinking, and therefore pedagogies that encourage critical thinking, such as case-based discussions, video tutorials, debates, and role-playing, would be more suitable for information ethics curriculum. Additionally, in terms of the nature of the learning resources, given the many theoretical frameworks of information ethics proposed by previous research (Hunt and Vitell, 1986; Thong and Yap, 1998), the transformation of these conceptual norms into practical guidelines of actions and curriculum calls for further efforts to clearly structure the elements of information ethics. Furthermore, information professional organizations in different fields follow different codes of ethics due to different backgrounds, field knowledge, and even the content of their pre-service training. For example, the professional training objectives of the library and information science, computer science, and engineering fields are aligned with those of information technology, but the focus of education for library and information science personnel is on the professional knowledge and information application skills required to provide library and information services (ASIS&T, 1992; IFLA, 2012; ALA, 2021), with an emphasis on people and services. Those in the information technology field are more oriented toward learning how to apply various information technology techniques (ACM, 2018; IEEE, 2020), and they thus focus on the data and technology itself. In addition to the differences in the professional training received in school, the actual practices and professional ethics of the workplace are also different in the field of library and information science and the field of information technology. Therefore, the scope of information professionals should be expanded to include other professionals in the field of library and information science and information technology, teachers and students, in addition to the previous focus on management information system engineers or professionals, in order to truly meet the contemporary social context.

Motivated by the aforementioned issues, the purpose of this study was to explore the key factors that influence the decision-making behaviors of information professionals when they are faced with information ethics issues. In addition, this study employed the professional field of library and information science and the field of information technology as examples to compare whether information professionals in different fields make different judgments on the aforementioned decision-making criteria, and then it explored the possible influencing factors and the planning of information ethics education for professionals. Past empirical studies of ethics were often framed around dilemmas, mostly in the form of situational questions for which examinees would have to make the choice they thought more appropriate (often one out of two). However, the purpose of this study was not to directly assess the subjects’ ethical decision making choices regarding the stated situational issues by means of situational questions, but rather to collate the guidelines suggested by professional organizations and then to allow professionals to assess the relative importance of these guidelines.

Therefore, this study focused on decision analysis as a theoretical and methodological approach which can be mainly applied to decision making problems under uncertainty and with several evaluation criteria, with reference to the Analytic Hierarchy Process (AHP) method developed to systematize complex problems, decompose them at different levels, and evaluate them in a comprehensive manner by quantifying them and finding their context (Saaty, 1990). This study first compiled the professional ethical standards developed by the major information professional societies and summarized the important elements of information ethics. The results of the study are intended to validate the professional information ethics standards compiled in the preceding section, and then to provide a reference contribution to the field of information ethics research by providing information on the aspects that need attention in the cultivation of professionals in different fields.

## Information ethics for information professionals

Ethics are a set of principles and concepts that judge whether a behavior is right or not. Compared to morals, ethics focus on the complex relationships and interactions between people at the social level. Various forms of ethical theories have been discussed and developed for people to explore how to interact properly with others (Arnold and Bowie, 2019). Among them, four types of theoretical approaches, including consequence-based theories, duty-based theories, rights-based theories, and virtue-based theories, have been regarded as critical and fundamental in information ethics education (Fallis, 2007). These four approaches complement one another by their alternative emphases on different aspects of information ethics, and mastering them facilitates learners’ further understanding of the importance of the ethical codes or principles. Furthermore, to adopt these approaches in the ethical decision-making process, Thong and Yap (1998) in their empirical investigation of 243 entry-level information system professionals suggested consequence-based approaches that reveal the consequences of ethical/unethical behaviors, which could facilitate learners’ comprehension of the codes and were more likely to lead to ethical decisions.

As information ethics involves field practices and interaction with others for common well-being, practical concerns have placed great emphasis on issues related to information ethics in the contexts of different professionals, types of information, and rapidly changing technologies. Since the 1980s, several professional societies of informaticists, such as the Association for Computing Machinery (Gotterbarn et al., 2018), Institute of Electrical and Electronics Engineers (IEEE, 2020), American Library Association (Witt, 2017; ALA, 2021), and International Federation of Library Associations (IFLA, 2012; Trepanier et al., 2019) have developed their own codes of ethics to guide practices of handling information ethics issues. These codes of ethics demonstrate the discipline-specific values and responsibilities of information professionals and institutions to society, as library-related associations accentuated ethical issues in providing information services (LAROC, 2002; IFLA, 2012; ALA, 2021) while computer societies highlighted concerns about producing information (ASIS&T, 1992; ACM, 2018; IEEE, 2020). However, as shown in Table 1, they also shared a common focus on issues raised by Mason (1986), whose systematic arguments about the four ethical issues of privacy, accuracy, property, and accessibility have attracted informaticists’ attention.

**TABLE 1:** PAPA-related statements in professional codes of informaticists.

Library and information science		Computer science	
Institute	Related articles	Institute	Related articles
**Privacy**			
ALA	3. *We protect each library user’s right to privacy and confidentiality with respect to information sought or received and resources consulted, borrowed, acquired or transmitted.*	ACM	1.6 *Respect privacy* 1.7 *Honor confidentiality*
IFLA	3. *Privacy, secrecy and transparency: Librarians and other information workers respect personal privacy, and the protection of personal data, necessarily shared between individuals and institutions*…	IEEE	1. …*to strive to comply with ethical design and sustainable development practices, to protect the privacy of others, and to disclose promptly factors that might endanger the public or the environment.*
LAROC	10. *Librarians shall strictly observe business secrets, protect the privacy of readers, and not seek to benefit themselves or harm others.*	ASIS&T	*- To resist all forms of censorship, inappropriate selection and acquisitions policies, and biases in information selection, provision and dissemination*
**Accuracy**			
ALA	1. *We provide the highest level of service to all library users through appropriate and usefully organized resources; equitable service policies; equitable access; and accurate, unbiased, and courteous responses to all requests*	ACM	1.3 *Be honest and trustworthy.* 2.9 *Design and implement systems that are robustly and usably secure.*
IFLA	5. *Neutrality, personal integrity and professional skills: Librarians and other information workers are strictly committed to neutrality and an unbiased stance regarding collection, access and service*…	IEEE	5. *to seek, accept, and offer honest criticism of technical work, to acknowledge and correct errors, to be honest and realistic in stating claims or estimates based on available data*…
LAROC	3. *Based on the principle of neutrality, librarians should collect all kinds of information and protect the readers’ rights.*	ASIS&T	– *not knowingly making false statements or providing erroneous or misleading information* – *to improve the information systems*… *to the best of their means and abilities by providing the most reliable and accurate information and acknowledging the credibility of the sources as known or unknown*
**Property**			
ALA	4. *We respect intellectual property rights and advocate balance between the interests of information users and rights holders.*	ACM	*1.5 Respect the work required to produce new ideas, inventions, creative works, and computing artifacts.*
IFLA	4. *Open access and intellectual property:*…*Librarians and other information workers have a professional duty to advocate for exceptions and limitations to copyright restrictions for libraries*…	IEEE	4. *to avoid unlawful conduct in professional activities.* 5. …*And to credit properly the contributions of others*
LAROC	*4. Librarians should strive to preserve various kinds of library information and promote cultural exchange*	ASIS&T	– *not using their position beyond their authorized limits or not using their credentials to misrepresent themselves*
**Accessibility**			
ALA	1. *We provide the highest level of service to all library users through appropriate and usefully organized resources; equitable service policies; equitable access; and accurate, unbiased, and courteous responses to all requests.* 7. *We distinguish between our personal convictions and professional duties and do not allow our personal beliefs to interfere with fair representation of the aims of our institutions or the provision of access to their information resources.*	ACM	1.4 *Be fair and take action not to discriminate.* 2.7 *Foster public awareness and understanding of computing, related technologies, and their consequences*.
IFLA	2. *Responsibilities toward individuals and society:*…*librarians and other information workers ensure that the right of accessing information is not denied and that equitable services are provided for everyone*… 4. *Open access and intellectual property: Librarians and other information workers’ interest is to provide the best possible access for library users to information and ideas in any media or format*…	IEEE	2. *to improve the understanding by individuals and society of the capabilities and societal implications of conventional and emerging technologies, including intelligent systems.* 7. *to treat all persons fairly and with respect, and to not engage in discrimination based on characteristics such as race, religion, gender, disability, age, national origin*…
LAROC	2. *Librarians should provide services based on the principle of equal access and not discriminate.* 6. *Librarians should continue to improve readers’ access and ability to use library resources.*	ASIS&T	– *adhering to principles of due process and equality of opportunity.*

Our review in Table 1 shows that Mason’s four issues have been included in the professional ethics in both fields, suggesting a common emphasis on privacy, accuracy, property, and accessibility across information professionals’ practices. Nevertheless, due to the highly contextual nature of information ethics (Nissenbaum, 2019), even when the code of ethics is fully understood, the actual decisions and practices in the field could be much more complex and dilemmatic (Fallis, 2007; McMenemy et al., 2014). Advancing from the above analysis, Table 2 provides a further comparison and discussion of the three main criteria included in the two domains using Mason’s (1986) PAPA framework: general moral imperatives, specific professional responsibilities, and organizational/workplace responsibilities. These were categorized by considering the professional organizations’ information management norms and personnel roles in relation to their cognitive and behavioral performance. The four aforementioned issues are included in the following behavioral levels, which are covered in the content of the evaluation items.

**TABLE 2:** Comparison of the professional ethics criteria.

Library and information science	Computer science

Definition/Discussion	Definition/Discussion
**Main Characteristic 1: General moral imperatives**	
*The code of ethics in the field of library and information science is closely related to its purpose of promoting social, cultural and economic well-being, and is based on the Universal Declaration of Human Rights, which states that all human beings have the freedom of opinion, expression and access to information (IFLA, 2012).*	*The Code of Information Ethics, developed by professional associations in the field of information technology, takes into account the impact of information technology on society and aims to promote human and social well-being and avoid harming others. When the ACM updated its code of ethics in 2018, it further emphasized the importance of the public nature of IT, that all people are stakeholders in IT, that transparency should be promoted and that stable and trustworthy systems should be established, and that it is prudent to modify or discontinue infrastructure and functions that the public relies on (ACM, 1992, 2018).*
**Main Characteristic 2: Specific professional responsibilities**	
*Professional ethical judgments regarding the development of professional work and competencies, in the fields of both library and information science and information technology, are referred to in order to maintain the quality, effectiveness, and dignity of professional work, as well as to further and maintain one’s professional competencies, including the study of relevant legal issues.*	*The ACM Code of Conduct ACM (1992, 2018) more comprehensively states that one should understand the laws related to professional work, accept and provide appropriate professional commentary, respect contractual obligations and agreed liabilities, and be authorized to access computer and communication resources. In 2018, the association added details on how one should measure one’s professional competence.*
**Main Characteristic 3: Organizational/workplace responsibilities**	
*The Code of Ethics of the two professional associations covers fewer levels of professional ethical judgment on organizational responsibility and only includes the need to maintain the confidentiality of the organization’s mission.*	*In addition to the aforementioned role in maintaining the confidentiality of an organization’s mission, the field of information technology encompasses the use of computers and communication software that should be used under authorization. However, because of the importance of professional ethics for organizations, how to manage information is also gaining attention in information management issues (Buchanan et al., 2009).*
*Depending on the format of the information, the career stage, and the four issues of PAPA, it can be expanded as follows: protecting the privacy of the organization, protecting the confidentiality of products and services, and managing the accuracy of the data.*	

Based on the above analysis, the content of ethics for information professionals includes moral responsibility, professional ethics, and workplace ethics, while the content of the professional code of conduct in the field of library information implies the concept of a limited field in the library and is therefore closely integrated with the mission and values of the organization. The professional codes of conduct in the field of information technology tend to provide sets of principle-based guidelines. Among them, ACM’s Code of Ethics, released in 1992 (ACM, 2018), covers a wide range of topics and is regarded as a solid basis for considering informaticists’ practices. Therefore, it was used as the main framework of the hierarchical analysis and the development of assessment criteria in this study in an attempt to understand the importance of the different target constructs and assessment criteria for information professionals in the fields of both library and information science and information technology.

## Methodology

### The analytical hierarchy process method

This study adopted the Analytical Hierarchy Process (AHP), which is a Multiple Attribute Decision Making (MADM) method (Saaty, 1987). The behaviors of individuals and groups in making decisions are complex and often include many different dimensions of consideration (Lin and Huang, 2013). Because of the different characteristics of each decision-maker, the importance of each aspect in the decision-making process could also vary. Based on a review of the professional and information ethics literature in general, and specifically the ACM Codes of Ethics, this study first developed an analytical hierarchy of information ethics.

**FIGURE 1:**
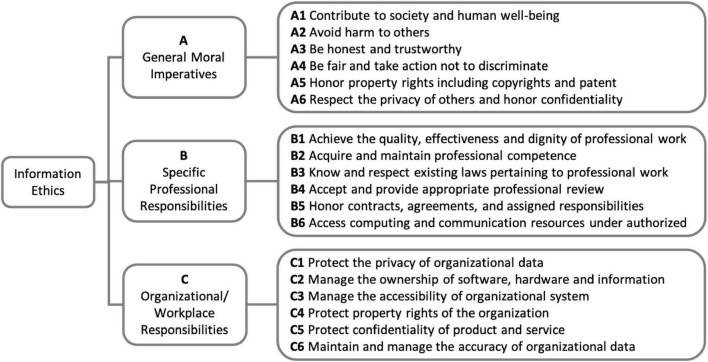
The analytical hierarchy of information ethics.

The concept of information ethics was composed of three major elements: general moral imperatives, specific professional responsibilities, and organizational/workplace responsibilities. With the three major elements as the main criteria for decision-making, sub-criteria were further developed as specific combinations of the characteristics of each main criterion. After a pilot study with 5 experts from academia and practice sectors of information science, several constructs under each major element were merged or deleted according to the rules of the AHP method by the value of consistency index and consistency ratio (Saaty, 1987). The final analytical hierarchy of information ethics is shown as Figure 1.

### Data collection and analysis

Based on the structure of the decision criteria, the final AHP questionnaire was compiled as a scale by Saaty (1987). This scale consists of all the criteria in Figure 1 and contains pairwise comparisons of criteria at each hierarchical level. The ranking system is on a scale from 1 to 9, where 1 means that two criteria are of equal importance and 9 indicates the highest importance of one criterion over the other. With the instrument, data were collected from experts with experience as practitioners and researchers in information science. They were drawn from departments or divisions of information science, information management, and information engineering in universities and in the public and private sectors that provide training, products, or services. These experts were invited to examine and weigh the critical factors regarding information ethics in the context of their practices with this AHP questionnaire. In this study, the data were analyzed in the Expert Choice 11.5 software package. After the model of the ethical hierarchy of information professionals was established, the judgment results of each expert’s answers were entered into a relative comparison matrix, after which the weights were calculated and the consistency of each questionnaire was checked.

## Results and discussions

### Analytic hierarchy process consistency testing

A total of 52 valid questionnaires were collected. The respondents included 25 IT experts (10 academic experts and 15 practitioner experts) and 27 library and information science experts (7 academic experts and 20 practitioner experts). Because consistency is essential for pairwise comparison matrices to ensure that the results are meaningful and it is also required by the AHP method (Xu and Xu, 2020), in order to ensure the validity of the pairwise comparisons and achieve uniformity of responses, each questionnaire was checked for consistency (Saaty, 1987). Methodologically, the consistency index (CI) of the derived weights should be less than 0.1 to ensure that the set of judgments is reliable. The analysis of the overall inconsistency index (O.I.I) suggested that 10 responses out of 25 IT experts and 7 responses out of 27 LIS experts should be removed due to insufficient consistency (O.I.I. > 0.1).

As shown in Table 3, the final number of respondents included in the AHP analysis was 35, including 15 experts in the IT field (8 academic experts and 7 practitioner experts) and 20 experts in the library and information science field (5 academic experts and 15 practitioner experts). The overall inconsistency index (O.I.I.) for both fields was less than 0.1, indicating that the hierarchical structure was appropriate for comparative analysis of subsequent levels of decision-making elements.

**TABLE 3:** Background information of the respondents providing valid responses.

Field	Academic	Practitioner	Total
Information Technology (IT)	8	7	15
Library and Information Science (LIS)	5	15	20
Total	13	22	35

### The main criteria analysis of two expert groups

The main criteria of “general moral imperatives,” “specific professional responsibilities,” and “organizational/workplace responsibilities” valued by different informaticist groups of information technology (IT) and library and information science (LIS) are listed in Tables 4, 5, respectively. The results suggested that, although both the academic and practitioner IT experts were identical in their emphasis on the three criteria, academic experts in LIS held views that were in almost direct opposition to those of their practitioner peers. The practitioner experts in IT and LIS areas ranked the three main criteria identically as follows: “general moral imperatives” was the most important, followed by “organizational/workplace responsibilities” and then “specific professional responsibilities.” The academic IT experts possessed the same emphasis as the practitioner, but the academic LIS experts regarded organizational/workplace responsibilities as the most important. The results implied the different impacts of the technical and contextual features of information service on decision-making. While technical characteristics were applicable across organizations, contextual factors could mostly vary from organization to organization. Therefore, academic experts in the LIS area, who often needed to consider the overall library service across institutions, could place more emphasis on individual organizational/workplace factors. This is also in line with previous studies that have found that LIS professionals have more ethical issues and considerations due to the human-centered nature of their discipline and professional conduct (Fallis, 2007; ALA, 2021; Lin et al., 2022).

**TABLE 4:** Three main criteria weights and rankings by experts in the IT field.

Main criteria	C.I.		Weights		Priority	
	Academic	Practitioner	Academic	Practitioner	Academic	Practitioner
General Moral Imperatives	0.01	0.03	0.491	0.606	1	1
Specific Professional Responsibilities	0.02	0.02	0.192	0.166	3	3
Organizational/workplace Responsibilities	0.009	0.03	0.317	0.228	2	2

In the aspect, the academic experts’ C.I. was 0.00057; the practitioners’ C.I. was 0.00126.

**TABLE 5:** Three main criteria weights and ranking by experts in the LIS field.

Main criteria	C.I.		Weights		Priority	
	Academic	Practitioner	Academic	Practitioner	Academic	Practitioner
General Moral Imperatives	0.03	0.00593	0.349	0.556	2	1
Specific Professional Responsibilities	0.02	0.00614	0.245	0.193	3	3
Organizational/workplace Responsibilities	0.01	0.00581	0.406	0.251	1	2

In this aspect, the academic experts’ C.I. was 0.0011; the practitioners’ C.I. was 0.00008.

In addition, all respondents considered “specific professional responsibilities” to be the least important, indicating that both academic experts and practical experts mostly considered the critical component of ethics that affected information personnel to be general moral imperatives. It is possible that, when people face ethical dilemmas, the first consideration is the intrinsic value judgment accumulated from past experiences which shaped their moral cognition.

### Analysis of the general moral imperatives criteria of two expert groups

For the analysis of the second level of the hierarchy, six evaluation sub-criteria were included in the general moral imperatives criteria, and the results of the evaluation are shown in Tables 6, 7. IT academics, practitioners, and LIS academic experts all ranked “avoid harm to others” as the most important criterion for assessing general moral imperatives. This criterion was also ranked second only to “respect the privacy of others and honor confidentiality” by the LIS practitioner experts. The least important criterion was “Contribute to society and human well-being,” which was ranked sixth by all the experts except the practitioners in the IT field, who ranked it fourth.

**TABLE 6:** Weights and ranking of general moral imperatives criteria by IT experts.

Criteria	Weights		Priority	
	Academic	Practitioner	Academic	Practitioner
**A1** Contribute to society and human well-being	0.069	0.134	6	4
**A2** Avoid harm to others	0.319	0.297	1	1
**A3** Be honest and trustworthy	0.198	0.213	2	2
**A4** Be fair and take action not to discriminate	0.160	0.098	3	6
**A5** Honor property rights including copyrights and patents	0.116	0.117	5	5
**A6** Respect the privacy of others and honor confidentiality	0.138	0.142	4	3

In this aspect, the academic experts’ C.I. was 0.03; the practitioners’ C.I. was 0.03.

**TABLE 7:** Weights and ranking of general moral imperatives criteria by LIS experts.

Criteria	Weights		Priority	
	Academic	Practitioner	Academic	Practitioner
**A1** Contribute to society and human well-being	0.095	0.080	6	6
**A2** Avoid harm to others	0.233	0.193	1	2
**A3** Be honest and trustworthy	0.203	0.143	2	5
**A4** Be fair and take action not to discriminate	0.159	0.166	4	4
**A5** Honor property rights including copyrights and patents	0.133	0.192	5	3
**A6** Respect the privacy of others and honor confidentiality	0.177	0.226	3	1

In this aspect, the academic’s C.I. was 0.03; the practitioners’ C.I. was 0.00593.

In addition, the results showed that the practitioners in the field of LIS gave more importance to the financial value and added value of the products or intellectual property protected by property rights, copyrights, patents, and intellectual property rights, and further rated the criteria as a higher priority, while the criterion of “Honor property rights including copyrights and patents” was ranked 5th by the other three groups of experts and not given much importance.

### Analysis of the specific professional responsibilities criteria of two expert groups

The specific professional responsibilities criteria contained six evaluation sub-criteria. As shown in Tables 8, 9, “achieve quality, effectiveness, and dignity of professional work,” “honor contracts, agreements, and assigned responsibilities,” and “access computing and communication resources with authorization” were all ranked in the top three in terms of importance by both academic and practitioner experts in the IT and LIS fields. The importance ranking of academic experts and practitioner experts in the LIS field was the same, from highest to lowest, for “honor contracts, agreements, and assigned responsibilities”, “access computing and communication resources with authorization,” and “achieve quality, effectiveness, and dignity of professional work,” respectively.

**TABLE 8:** Weights and ranking of specific professional responsibilities criteria by IT experts.

Criteria	Weights		Priority	
	Academic	Practitioner	Academic	Practitioner
**B1** Achieve the quality, effectiveness and dignity of professional work	0.179	0.218	3	1
**B2** Acquire and maintain professional competence	0.124	0.140	5	4
**B3** Know and respect existing laws pertaining to professional work	0.173	0.138	4	5
**B4** Accept and provide appropriate professional review	0.084	0.114	6	6
**B5** Honor contracts, agreements, and assigned responsibilities	0.203	0.214	2	2
**B6** Access computing and communication resources with authorization	0.237	0.176	1	3

In this aspect, the academic experts’ C.I. was 0.02; the practitioners’ C.I. was 0.02.

**TABLE 9:** Weights and ranking of specific professional responsibilities criteria by LIS experts.

Criteria	Weights		Priority	
	Academic	Practitioner	Academic	Practitioner
**B1** Achieve the quality, effectiveness and dignity of professional work	0.142	0.172	3	3
**B2** Acquire and maintain professional competence	0.102	0.105	5	6
**B3** Know and respect existing laws pertaining to professional work	0.131	0.155	4	4
**B4** Accept and provide appropriate professional review	0.099	0.134	6	5
**B5** Honor contracts, agreements, and assigned responsibilities	0.266	0.230	1	1
**B6** Access computing and communication resources with authorization	0.259	0.205	2	2

In this aspect, the academic experts’ C.I. was 0.02; the practitioners’ C.I. was 0.00614.

In addition, academic experts in both domains ranked the importance of the “specific professional responsibilities” criteria in roughly the same order. Only the academic experts in the IT field considered “access computing and communication resources with authorization” to be more important than “honor contracts, agreements, and assigned responsibilities,” while the academic experts in the LIS field thought the opposite. It was inferred that the work nature and environment of the academic experts were similar, so the priority of the evaluation criteria for “specific professional responsibilities” related to the workplace would be almost the same.

### Analysis of the organizational/workplace responsibilities criteria of two expert groups

The organizational/workplace responsibilities criteria contained six evaluation sub-criteria. As shown in Tables 10, 11 below, “protect the privacy of organizational data” was the most important criterion for experts in both fields. The next most important criteria were more divergent and mainly included “protect confidentiality of products and services,” “protect property rights of the organization,” and “maintain and manage the accuracy of organizational data.” The criteria of “manage the ownership of software, hardware and information” and “manage the accessibility of the organizational system” were ranked lowest.

**TABLE 10:** Weights and ranking of organizational/workplace responsibilities criteria by IT experts.

Criteria	Weight		Priority	
	Academic	Practitioner	Academic	Practitioner
**C1** Protect the privacy of organizational data	0.250	0.344	1	1
**C2** Manage the ownership of software, hardware and information	0.148	0.048	3	6
**C3** Manage the accessibility of the organizational system	0.122	0.077	6	5
**C4** Protect property rights of the organization	0.140	0.182	5	2
**C5** Protect confidentiality of products and services	0.147	0.182	4	2
**C6** Maintain and manage the accuracy of organizational data	0.193	0.166	2	4

In this aspect, the academic experts’ C.I. was 0.02; the practitioners’ C.I. was 0.03.

**TABLE 11:** Weights and ranking of organizational/workplace responsibilities criteria by LIS experts.

Criteria	Weight		Priority	
	Academic	Practitioner	Academic	Practitioner
**C1** Protect the privacy of organizational data	0.275	0.212	1	1
**C2** Manage the ownership of software, hardware and information	0.122	0.096	6	6
**C3** Manage the accessibility of the organizational system	0.148	0.133	3	5
**C4** Protect property rights of the organization	0.136	0.178	4	4
**C5** Protect confidentiality of products and services	0.191	0.190	2	2
**C6** Maintain and manage the accuracy of organizational data	0.128	0.190	5	2

In this aspect, the academic experts’ C.I. was 0.01; the practitioners’ C.I. was 0.00581.

Possible explanations for the aforementioned evaluation results could be that IT academic experts were more management-oriented (García-Holgado et al., 2021), so they focused on the protection of the privacy of the organization’s products and services and emphasized the need to maintain the accuracy of management information. As for the IT practitioners, they adopted the perspective of employees in the organization, so the privacy of employee data, customer data, and vendor data that they dealt with every day was most important to them (D’Arcy and Devaraj, 2012; Lu et al., 2018), followed by the intellectual property rights of organizational resources, such as internal training materials and online databases (Yueh et al., 2016).

### The criteria analysis of information professionals in information technology and library and information science fields

This study further integrated the results of the AHP analysis between academic experts and practitioner experts in the two professional fields. As shown in Table 12, the criterion of “general moral imperatives” was given the highest priority in both the IT and LIS fields, followed by “organizational/workplace responsibilities” and finally “specific professional responsibilities.”

**TABLE 12:** Weights and ranking of three main criteria by information professionals in IT and LIS fields.

Aspects	C.I.		Weight		Priority	
	IT	LIS	IT	LIS	IT	LIS
General Moral Imperatives	0.01	0.00874	0.545	0.503	1	1
Specific Professional Responsibilities	0.00967	0.00499	0.181	0.208	3	3
Organizational/workplace Responsibilities	0.00918	0.00401	0.274	0.288	2	2

In this aspect, the IT expert’s C.I. was 0.000086; the LIS expert’s C.I. was 0.00001.

In addition to the above three main criteria weights, the product of the main criteria weights and the sub-criteria weights was further calculated to represent the global priority of each evaluation criterion, and the relative weight rankings of all evaluation criteria were obtained as shown in Tables 13, 14.

**TABLE 13:** Information technology experts’ local and global weights of all criteria of the hierarchy.

	Aspects	Criteria
Information Ethics	General Moral Imperatives	**A1** Contribute to society and human well-being (L:0.095; G:0.052)
	(L:0.545)	**A2** Avoid harm to others (L:0.310; G:0.169)
		**A3** Be honest and trustworthy (L:0.208; G:0.113)
		**A4** Be fair and take action not to discriminate (L:0.128; G:0.070)
		**A5** Honor property rights including copyrights and patents (L:0.118; G:0.064)
		**A6** Respect the privacy of others and honor confidentiality (L:0.141; G:0.077)
	Specific Professional	**B1** Achieve the quality, effectiveness and dignity of professional work (L:0.196; G:0.036)
	Responsibilities (L:0.181)	**B2** Acquire and maintain professional competence (L:0.133; G:0.024)
		**B3** Know and respect existing laws pertaining to professional work (L:0.157; G:0.028)
		**B4** Accept and provide appropriate professional review (L:0.098; G:0.018)
		**B5** Honor contracts, agreements, and assigned responsibilities (L:0.210; G:0.038)
		**B6** Access computing and communication resources with authorization (L:0.206; G:0.037)
	Organizational/workplace	**C1** Protect the privacy of organizational data (L:0.296; G:0.081)
	Responsibilities (L:0.274)	**C2** Manage the ownership of software, hardware and information (L:0.090; G:0.025)
		**C3** Manage the accessibility of the organizational system (L:0.100; G:0.027)
		**C4** Protect property rights of the organization (L:0.162; G:0.044)
		**C5** Protect confidentiality of products and services (L:0.167; G:0.046)
		**C6** Maintain and manage the accuracy of organizational data (L:0.185; G:0.051)

**TABLE 14:** Library and information science experts’ local and global weights of all criteria of the hierarchy.

	Aspects	Criteria
Information Ethics	General Moral Imperatives	**A1** Contribute to society and human well-being (L:0.084; G:0.042)
	(L:0.503)	**A2** Avoid harm to others (L:0.203; G:0.102)
		**A3** Be honest and trustworthy (L:0.157; G:0.079)
		**A4** Be fair and take action not to discriminate (L:0.166; G:0.083)
		**A5** Honor property rights including copyrights and patents (L:0.176; G:0.089)
		**A6** Respect the privacy of others and honor confidentiality (L:0.214; G:0.108)
	Specific Professional	**B1** Achieve the quality, effectiveness and dignity of professional work (L:0.164; G:0.034)
	Responsibilities (L:0.208)	**B2** Acquire and maintain professional competence (L:0.104; G:0.022)
		**B3** Know and respect existing laws pertaining to professional work (L:0.149; G:0.031)
		**B4** Accept and provide appropriate professional review (L:0.124; G:0.026)
		**B5** Honor contracts, agreements, and assigned responsibilities (L:0.240; G:0.050)
		**B6** Access computing and communication resources with authorization (L:0.219; G:0.046)
	Organizational/workplace	**C1** Protect the privacy of organizational data (L:0.227; G:0.065)
	Responsibilities (L:0.288)	**C2** Manage the ownership of software, hardware and information (L:0.102; G:0.029)
		**C3** Manage the accessibility of the organizational system (L:0.138; G:0.040)
		**C4** Protect property rights of the organization (L:0.168; G:0.048)
		**C5** Protect confidentiality of products and services (L:0.191; G:0.055)
		**C6** Maintain and manage the accuracy of organizational data (L:0.174; G:0.050)

After further comparing the 18 sub-criteria, this study found that the most important criterion ranked by IT experts was “avoid harm to others” (GW = 0.113), while the criterion ranked most important by LIS experts was “respect the privacy of others and honor confidentiality” (GW = 0.108), which was slightly higher than “avoid harm to others” (GW = 0.102). The results echoed the findings of the previous studies (Eskens, 2020; Zimmer et al., 2020), supporting that informaticists, like the general public, placed the highest value on privacy in information ethics issues.

Many past studies have argued that Orientalism or Asian culture, which is strongly influenced by Confucianism, has shaped certain Asian values, one of which is “interpersonal reciprocity and accommodation” (avoiding conflict with others) (Nathan, 2012). In Confucian relationships, interpersonal relationships that give special consideration to the law of human relations may create a dilemma of human relations leading to subjective decisions due to the law of equity (Huang, 2001). Therefore, people in this cultural context tend to avoid conflict (including in verbal and non-verbal communication), prevent hurting others, and take care not to offend others. The results of the professionals’ evaluations of these ethical criteria in this study may be related to the aforementioned characteristics of these cultural contextual influences.

## Conclusion

This study used the analytic hierarchical process method to develop an evaluation framework and criteria for information professional ethics, and it also attempted to understand the differences in the ranking of the importance of the criteria for evaluating information professional ethics between practitioners in field of the information technology and those in the field of library and information science. From the results of the study, it was found that the basic attitudes or perceptions of the practitioners in the two professional fields toward the ethical issues of information profession were similar, and both of them focused the most on the general moral imperatives aspect and the need to avoid hurting others or violating others’ privacy.

Furthermore, the analysis of the general moral imperatives suggested that expert respondents gave priorities to aspects that were directly and specifically related to personal interests. They were most concerned with causing harm to others and themselves, and more general aspects that caused no immediate harm were weighted less. The expert respondents’ evaluations of the specific professional responsibilities suggested that factors that directly related to their work were regarded as the most important. They also gave high priority to legal issues, since the codification of norms suggested clearer guidelines of action to follow. In the aspect of organizational/workplace responsibilities, factors involved with interests and conflicts with external institutions were given the most weight over the others, such as the protection of organizational information of clients, suppliers, and employees. On the other hand, internal exchange or circulation of information was regarded as less critical. Overall, the findings suggested that moral imperatives as personal traits were regarded as the most important, followed by personal competence in work and finally social interactions with others.

The results of this study can provide information professional organizations, academic researchers and practitioners with important references on information ethics issues. In particular, the results of this study will provide a deeper understanding of the decision-making behaviors of professionals in the face of ethical issues and serve as a reference for planning a comprehensive framework for professional training programs in information ethics education and training. In addition, the cultural contexts and personal characteristics involved in these ethical decisions are worthy of further exploration in future research. Despite considerations of disciplinary diversity, a few limitations of this study should be noticed. Since this study analyzed and compared only the results of subjective questionnaires completed by experts and scholars, their reasons and justifications for the rankings inferred by the researchers could still be insufficient to include all possibilities. Based on the findings of this study, future studies should be conducted to develop teaching materials and assessment tools for information ethics so as to help nurture talents in related information professional fields.

## Data availability statement

The raw data supporting the conclusions of this article will be made available by the authors, without undue reservation.

## Ethics statement

Ethical review and approval was not required for the study on human participants in accordance with the local legislation and institutional requirements. The patients/participants provided their written informed consent to participate in this study.

## Author contributions

H-PY: conceptualization, methodology, funding acquisition, and writing – original draft, review and editing. C-YH: investigation, formal analysis, and visualization. WL: methodology, investigation, validation, and writing – review and editing. All authors contributed to the article and approved the submitted version.
